# Evaluation of *Providencia rettgeri* pathogenicity against laboratory Mediterranean fruit fly strain (*Ceratitis capitata*)

**DOI:** 10.1371/journal.pone.0196343

**Published:** 2018-05-07

**Authors:** Meriem Msaad Guerfali, Wafa Djobbi, Kamel Charaabi, Heithem Hamden, Salma Fadhl, Wafa Marzouki, Ferjani Dhaouedi, Claude Chevrier

**Affiliations:** 1 Laboratory of biotechnology and nuclear technologies, LR16CNSTN01, National centre of nuclear sciences and technologies, Technopole Sidi Thabet, Tunis, Tunisia; 2 UMRCNRS, 6035, Insect Research Biology Institute (IRBI), Faculty of Science and Technology, Tours, France; University of Thessaly School of Agricultural Sciences, GREECE

## Abstract

The Mediterranean fruit fly (medfly) *Ceratitis capitata* (Wiedemann) (Diptera: Tephritidae), is often referred to as the most severe agricultural pest. Its biological control is mainly through the Sterile Insect Technique (SIT). Colonization, mass-rearing conditions and the irradiation process impact the competitiveness of sterile males and disrupt symbiotic associations by favoring some bacterial species and suppressing others. Levels of *Providencia* species have been shown to fluctuate considerably in the gut of the medfly laboratory strain Vienna 8 under irradiation, increasing by up to 22%. This study aimed to determine the pathogenicity of *Providencia rettgeri* isolated from the gut of laboratory Vienna 8 medfly strains by examining the effects of 1) two different treatment doses on egg-hatching and development and 2) two infection methodologies (ingestion and injection) of male and female adults according to their mating status. Treatment of eggs with *P*. *rettgeri* (2%) significantly decreased the mean egg to pupae recovery rate. Our data showed significant high mortality in flies with both injection and ingestion after 24 hours without any effect of sex. Microbial counts demonstrated that the bacteria could proliferate and replicate in adult flies. There was a significant sex-dependent effect after infection, with mortality decreasing significantly for males more than females. *Providencia rettgeri* can be considered as a potential pathogen of *C*. *capitata*. Mating protected males and females against infection by *P*. *rettgeri* by triggering an immune response leading to double the levels of *Cecropin* being secreted compared to infected virgin adults, thus reducing the virulence of the bacteria.

## Introduction

The Mediterranean fruit fly (medfly), *Ceratitis capitata* (Wiedemann) (Diptera: Tephritidae), is often referred to as the most sever agricultural pest [1,2] with a host range that exceeds 300 plant species. Although various techniques have been developed, medfly population control still relies on chemicals targeting the adult stage. However, public concern about pesticides use is increasing. Pesticides are not specific, they cause resistance within insect pests, have deleterious effects on human health and pollute water and air. In recent years, biological control of medfly has mainly involved the Sterile Insect Technique (SIT). SIT is a biological environmentally friendly method of pest suppression or elimination which fits very well to the area wide approach of integrated pest management (AW-IPM) [3]. Since the seventies, this technique has become widely applied against *C*. *capitata* populations. SIT is a species-specific technique that is based on mass production of the target species, its sterilization and release in the field. Sterile released males will copulate with wild virgin females, that will give unviable eggs and the wild population density collapses over time [4]. However, SIT success is mainly related to male fitness and competitiveness [5]. Nonetheless, these males have undergone different stresses such as colonization, mass rearing and irradiation.

These processes affect the produced males and disadvantage them compared to the wild ones [6, 7, 8, 9, 10, 11, 12, 13, 14]. Similarly, the irradiation process was reported to act on the structure of the microbial community in mass-reared males. For Vienna 8 flies, a mass reared genetic sexing strain of *C*. *capitata*, it remarkably changes the gut microbial composition of the males coming from the mass rearing in relation to the wild ones [15].

Enterobacteriaceae was reported to be the solely predominant bacterial taxon in field caught medflies gut [15, 16, 17]. The Enterobacteriaceae that frequently occurred are *Klebsiella* spp., *Pantoae* spp., *Enterobacter* spp., *Pectobacterium* spp., and *Citobacter freundii* [18, 19, 20]. Though, the diversity of Enterobacteriaceae in mass reared irradiated flies is significantly reduced giving an advantage to *Pseudomonas* species [15]. This change in structure is suspected to be the cause of the low competitiveness of these males, since it has been shown that the role of the gut microbial community is determinant in fitness and several traits of the history of the insect [18, 21,22].

Indeed, Behar and colleagues [18–23], and Ben Yosef and colleagues [24], suggested that the microbiota associated with the fly’s digestive tract may play a significant role in fitness during the various stages of the host’s life cycle.

The most notable example of reduction in the Enterobacteriaceae concerns the fluctuation of the species *Providencia* in the gut of medfly after irradiation. Ben Ami and colleagues [15] showed that on hatching *Providencia* sp. was prevalent at the rate of 9% in caught wild males and 12% in the laboratory strain Vienna 8. However, in flies irradiated, on hatching day *Providencia* sp. dropped to 4%, then at five days old it increased to 22%. Given that *Providencia* species exhibit pathogenicity against some Diptera, we can assume that these relatively high infection rates of up to 22% at five days of age could affect medfly fitness. Bacterial colonization of the insect’s midgut could be the cause of persistence and excessive proliferation resulting in harm to the insect [25].

*Providencia* is classified as a Gram-negative opportunistic, non-spore forming pathogens [26] that have been isolated from many insect orders. Numerous studies of bacteria associated with insects such as blow fly [27], stable fly [28], Mexican fruit fly [29], Olive fly [30] and the Mediterranean fruit fly [15], have isolated the bacteria either from the whole insect or specifically from the gut.

*Providencia* strains have also been isolated as infectious agents of *Drosophila melanogaster* and have been shown to have distinct phenotypes including different virulence towards *D*. *melanogaster* [31–32], with highly virulent phenotypes causing high mortality and weakly virulent phenotypes causing moderate mortality [32]. The most virulent bacteria proliferated within the infected flies and induced the expression of antibacterial immune genes [32]. However, they have shown that *P*. *sneebia* causing complete mortality is able to actively avoid detection by the immune system as well as protect itself from the immune response.

The pathology of *Providencia* species and strains was defined by Galac and Lazzaro [32] as the proportion of host mortality caused by the bacteria, its faculty to proliferate within the fly, and the levels of host immunity response triggered by infection, that is measured by the expression of antimicrobial peptide (APM) genes. Insects are able to activate their humoral immune system in response to bacteria through the production of antimicrobial peptides (AMPs), in addition to the cellular immune response. The AMPs allow the insect to cope with the infection by reaching significant concentrations in the hemolymph. Over the last decade, AMPs in medfly have been found and characterized and including *Cecropin* [33, 34, 35]. The pathology of *Providencia* can be highly variable according to the phenotype and insect species. For a given host and pathogen pair, bacterial proliferation and host mortality may or may not be correlated [31].

Previous studies have investigated the virulence of *Providencia* in many insect orders and its prevalence in a laboratory medfly strain destined for producing males with high fitness components for use in SIT. In the present study, we tried to determine the pathogenicity of *P*. *rettgeri* against medfly, by first treating eggs with two different doses and then assessing the effect on egg hatching and development. In a second stage, we examined the pathogenicity of *P*. *rettgeri* according to two methods of infection of male and female adults, 1) comparing the male and female adult mortality according to the infection method, 2) followed by the monitoring proliferation of bacteria and their ability to replicate in male and female adults. To do this end, we measured the bacterial load at multiple time points after infection according to injection method. Although pathogenicity has been reported for several insect species, most methods involved injection of bacteria into insects rather than ingestion of the bacteria by the insect. Ingestion is considered to have little consequence for insects [36]; but [37] demonstrated different results when using the same kind of bacteria (*Serratia marcescens*) in other insect species.

Immune defense, is defined as the combined ability of an organism to actively fight and tolerate infection [38]. This defense system is considered as an essential part of the energetic investment of single individuals leading to high fitness and fecundity costs [39–40]. It has been suggested that mating may have immunosuppressive effects in females, so that available resources are not dissipated in the induction of immunity, but instead, will be deployed towards the reproductive potential. Moreover, mating can cause decreased survival after infection with a pathogen in females [40], apart from post-mating immunosuppression may not be systematically activated. Therefore, in a third stage, to investigate the effect of mating status we infected mated males and females and measured their mortality and bacterial load at different post-infection times, and also quantified the relative density of cDNA amplicons of *Cecropin* gene expression.

## Materials and methods

### Fly stock and maintenance

The Mediterranean fruit flies were from a colony of the VIENNA 8 genetic sexing strain (GSS) maintained in the Tunisian Medfly rearing facility situated in the National Center for Nuclear Science and Technology (CNSTN). This strain has two mutations with two markers *wp* and *tsl* on Y-autosome 5 producing wild-type (brown pupae) males and mutant (white pupae) females [41]. Adult flies were fed with sugar:yeast (3:1) and water. The larval diet was based on the formulation originally suggested by Tanaka et al. [42]. It contained wheat bran as a bulking agent (28%), torula yeast as a protein source (7%), sugar as a phagostimulant and carbohydrate source (14%), and water (50%). All the experiments were carried out under laboratory conditions (23°C ±1°C and 60% RH).

### Isolation and identification of bacteria from the digestive tract of *Ceratitis capitata*

Three-day-old adult flies (n = 50) were dipped in a soap solution, then dipped rapidly in 70% ethanol for disinfection, and washed in sterile phosphate saline buffer (PBS) [20–21]. Their guts were sterilely extracted by dissection under a microscope and kept in 50μl of PBS. The extracted guts were homogenized using a sterile pestle, diluted at 10^−6^ in sterile water, and used to inoculate growth media. The diluted gut extract drops (100μl) were placed on CHROMagar orientation medium to isolate gram-negative bacilli according to the manufacturer’s instructions. Plates were incubated at 37°C. Bacterial colonies that developed were recorded 48 h later.

CHROMagar (CHROMagar ^™^, France) medium is a selective chromogenic medium based on color and morphology which provides 90 to 99% accuracy with additional simple biochemical tests such as indole, lysine, and decarboxylase and ornithine decarboxylase utilization tests. The *Providencia* isolate demonstrates diffuse brown halo colonies as a result of tryptophan deaminase production (TDA) [43–44].

The visible colonies were cultured for 24 hours to produce pure culture. Thereafter, they were identified using a commercially available phenotyping system API 20E (BioMerieux, Inc, France) which was used according to the manufacturer’s instructions.

### PCR amplification and analysis of the 16S *rDNA* sequence

After a primary identification of *Providencia rettgeri* further studies were conducted for improved accuracy. Universal primers (54-S-D-Bact-0008-a-S-20-34 and 54-S-D-Bact-1495-a-S-20-34) for amplification of the bacterial 16S *rDNA* gene were used [45] (S1 Table). The reaction mixture consisted of 10X PCR reaction buffer, 2.5 mM MgCl2, 0.12 mM deoxynucleoside triphosphate, 0.2 mM of each primer, 1 U Taq DNA polymerase and 1 ml of total DNA. The PCR program consisted of an initial step at 94°C for 3 min, 35 cycles of denaturation at 94°C for 45 s, annealing for 1 min at 55°C and elongation for 2 min at 72°C, followed by a final elongation step at 72°C for 8 min. The homology of the almost complete 16S *rDNA* gene sequence of the isolated strain was compared using the NCBI BLAST database with the BLAST algorithm (http://www.ncbi.nlm.nih.gov/).

### Bioassay of effect of *Providencia* sp. on *Ceratitis capitata* eggs

Newly laid eggs were collected in vials. Samples of 100 eggs were put in 10 ml of *Providencia* sp. suspension diluted (A600 = 1.0 ± 0.05) at 1 and 2% for 48 hours. Three replicates were carried out for both treated and non-treated eggs used as a control. Trays containing 100 gm of larva diet based on a bran substrate [42] were seeded with treated and non-treated eggs. The egg to pupae recovery was recorded for each treatment and the number of males (brown) and female (White) pupae was counted.

### Bioassay of effect of *Providencia* sp. at adult stage

#### Infection by injection

Flies (3–6 days old) were alternately anesthetized in groups of 10 under nitrogen. They were infected by dipping a 0.15 mm anodized steel needle (Fine Science 99 Tools, Inc.) into a dilute bacteria culture of the Gram-negative bacterial pathogen *P*. *rettgeri*, which was then used to pierce the thorax of the fly [32]. The *P*. *rettgeri* had previously been grown with shaking overnight in liquid Luria broth (LB) at 37°C from a single bacterial colony, then diluted in sterile LB to an optical density of A600 = 1.0 ± 0.05. A subset of flies n = 10 was pricked with a sterile needle as a wounding control (control Injection). Mortality was recorded 48 h post-infection. Five replicates were carried out for each group of flies. To assess the delivered infection dose, a subset of flies was transferred each, to a single microcentrifuge tube for analyses. The delivered bacterial concentration was ranging between 5 x10 ^2^ and 5x10 ^3^ bacteria per fly.

#### Infection by ingestion

This test was also carried out by adding 100 μl of the bacterial culture (A600 = 1.0 ± 0.05) to a piece of cotton wool instead of water. Ten flies are placed in a large Petri dish with food and the piece of cotton wool. For the control flies the cotton wool is imbibed with 100 μl of LB free of bacterial culture (control Ingestion). Prior to this, flies had been kept in clean cages without any food for 24h [46]. Mortality was recorded 48 h post-infection. Five replicates were set up for each treatment.

### Mating procedure

Male and female flies were collected as virgins three days post-emergence. They were lightly anesthetized with nitrogen and put into individual vials with *ad libitum* access to food. Females were allowed to recover overnight. The day before mating, a single virgin male was aspirated into each vial containing a female assigned to the “virgin” and individual copulations were observed. Males were removed from the females shortly after the end of copulation to prevent additional courting or copulation attempts. Females from copulations lasting for less than 15 min were discarded and not used for infections to maximize the likelihood that all females used in the experiment received a full complement of sperm and seminal fluid from their mates [32]. Fifteen males and 15 females were infected by injection 2.5 h after mating as described above. Fifteen virgin males flies and 15 virgin females were pricked with a sterile needle as a wounding control (S1 Fig). Five replicates were set up for each treatment. All replicates are carried out from flies coming from the same flies generation for the sections of infection by injection, infection by ingestion and mating procedure.

### Bacterial load assay

To assay bacterial load, each infected fly with *P*. *rettgeri* by injection and by ingestion is set in a single microcentrifuge tube and homogenized. The homogenate was diluted and 50 μl was deposited in a spiral pattern on LB agar at 0 and 24 h after infection. Plates were incubated overnight at 37°C [32]. Most bacteria used for pathogenic infection grow much faster than the gut microbiota on LB agar at 37 °C. The grown colonies are counted with an automated colony counter to assess the bacterial load of homogenate based on the number of colonies and their position. Three replicates for the control and for each time assay were set up.

### RNA extraction and reverse-transcriptase PCR

*Cecropins* are antibacterial peptides that are synthesized in insects as a response to infection. *Cecropin* transcripts appear within an hour of bacteria injection into the hemocoel and reach a maximum after 2–6 h [47].

We extracted total RNA with Trizol from three infected virgin and mated males and females with injection at 0h, 2h and 4h. RNA was then reverse transcribed to cDNA from poly-T primers using standard procedures [48]. The purity of RNA was verified by measuring absorbance at 260–280 nm; RNA with an absorbance ratio of 260–280 nm (>1.8) was used for the experiments. For each individual 200 ng of the extracted total RNA was transcribed into cDNA.

### Real-time quantitative PCR

The cDNA’s were used to assess the relative transcript abundance of the *Cecropin* gene. Two medfly reference genes (*GAPDH2* and *G6PDH*) were used for normalization. Real-time PCR was performed with the Super mix (Syber ^®^ Premix Ex Teq ^™^). Cycling parameters were: 3 minutes at 95°C, 40 cycles of 10 seconds at 95°C and 30 seconds at 57°C and 30 seconds at 68°C. They were performed using the Bio-Rad DNA Engine Mini Opticon real-time PCR detector and SYBR green dye. A fluorescence reading was made at the end of each extension step. Three replicates were performed and specificity of the amplification products was assessed by melt-curve analysis. PCR efficiencies were above 87% for all primer pairs. The primer pairs are as follow, X70030 (*Cecropin 1*) 5’ gcgggttggctgaagaag 3’ (Forward primer) 5’ cggtggctgcgacattag 3’ (Reverse primer); FS831 (*GAPDH2*) 5 ‘ggtcgcatcggtcgtctgg 3’ (Forward primer) 5’ gctgaaacggtgcccttgaaac 3’ (Reverse primer) and S67872 (*G6PDH*) 5’ cggacgagcaggcaaaatatg 3’ (Forward primer) and 5’ agacggacggcggtaagg 3’ (Reverse primer) [36, 49] (S1 Table). Experimental sample concentrations were derived from standard curves (S2A, S2B and S2C Fig). The test samples (males and females infected mated flies 0h, 2h and 4h) and the calibrator samples (males and females infected virgin flies 0h, 2h and 4h) were normalized by the geometric mean of the two reference genes. The fold-difference between the test samples and the calibrator samples is then obtained by the ratio (Normalized test sample/Normalized calibrator sample) as described at [http://www.appliedbiosystems.com].

### Statistical analyses

Each test was repeated five times, except for Real-Time quantification, where three replicates were performed. A control was set up for each experiment. For ingestion treatment, the control flies have been put in contact with a cotton imbibed with LB instead of bacterial culture (Control Ingestion). On the other hand, for injection treatment the control flies were sterilely wounded (Control Injection). All data are presented as means ± SE. Differences between groups were tested using one-way analysis of variance (ANOVA) and a multivariate analysis of variance (MANOVA) (Stat graphics Centurion XVI) using the data that had previously been checked for normality. Significant differences between groups were determined by a least significant difference (LSD) test at 95% CL. To test the correlations between mortality levels and the bacterial load, we used the hypothesis test in stat graphics Centurion XVI.

## Results

### Isolation and identification of bacteria from the digestive tract of *Ceratitis capitata*

The isolation was based first on colony color and morphology on CHROMagar medium (diffuse brown halo colony) and adjunctive biochemical tests confirmed the *Providencia* species as Indole-positive and Ornithine-negative. On a molecular basis, the isolated strain was identified as *P*. *rettgeri* after 16S *rDNA* gene sequence analyses (S3 Fig). The 16S*rDNA* gene sequence of 1485 bp showed 99% sequence identity with *P*. *rettgeri*, NR-115880.1 in GeneBank database under the accession number of MG752971 (S1 Table). This isolate is a representative of the three clades of *P*. *rettgeri* group according to *rpoB* clustering [50]. These results suggest that this isolate could be a new variant related to *C*. *capitata*.

### Egg hatch

**Fig 1:**
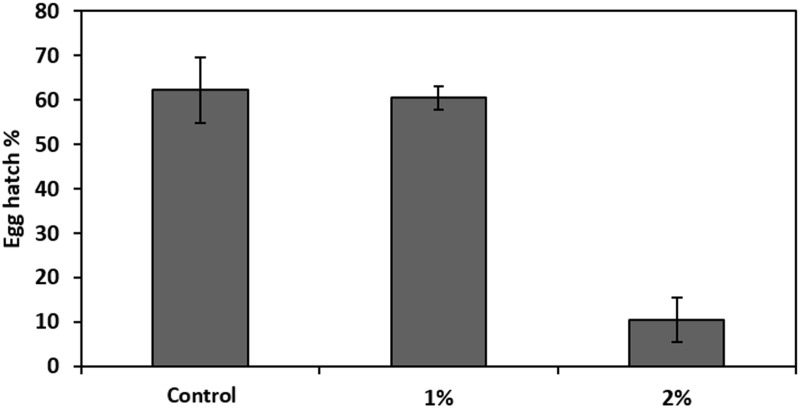
Effect of *Providencia rettgeri* (suspensions at 1 and 2%). Control corresponds to noninfected eggs. Each bar shows mean ± SE.

**Fig 2:**
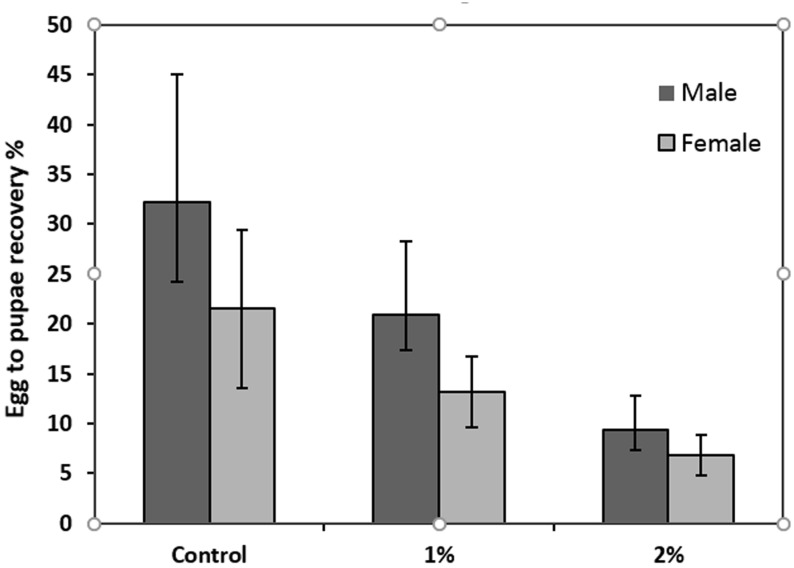
Effect of *Providencia rettgeri* (suspension at 1 and 2%) on egg to pupae recovery. Control corresponds to non infected eggs. Male represent brown pupae and Female represent white pupae. Each bar shows mean percentage ± SE.

The analyses revealed that infecting the eggs with *Providencia rettgeri* suspensions at 1 and 2% significantly decreased the egg hatch percentage compared to the control eggs that were suspended in water only (F = 30.29, df = 2, P<0.05) at the 95% confidence level (LSD 95% test) (Fig 1). For each treatment (1 and 2%) the total number of pupae collected was counted. The mean recovery for the control males (Brown pupae) and females (white pupae) obtained from eggs suspended in water only was (32.2±12.8 and 21.5±7.9, respectively) (Fig 2). The 2% suspension infection significantly decreased the mean recovery (F = 3.87, df = 2, P<0.05) compared to the control. The lowest value of recovery was obtained with a 2% infection for males and females (9.3±3.3 and 6.8±2, respectively). Sex had no significant effect on the recovery (F = 1.62, df = 2, P = 0.2).

### Survival assay after infection of *Ceratitis capitata* males and females with *Providencia rettgeri* and bacterial load proliferation

**Fig 3:**
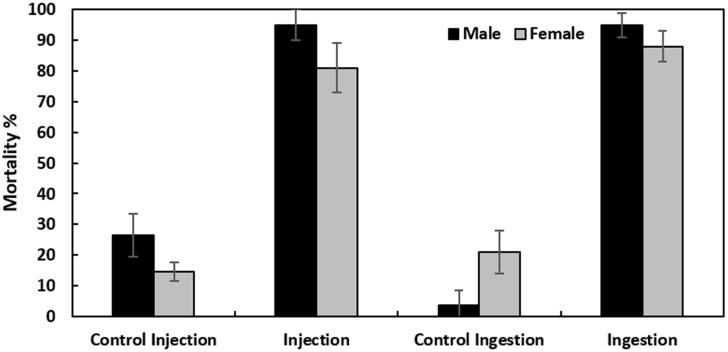
Mortality of male and female *Ceratitis capitata* following infection by *Providencia rettgeri* by injection or ingestion. Control Injection, represents flies (males and females) that were sterilely wounded and Control Ingestion, represents flies (males and females) that were in contact with cotton imbibed with LB only. Each bar shows mean percentage± SE.

In order to test the ability of *Providencia rettgeri* to cause mortality on adult *C*. *capitata*, virgin males and females were infected by injection and ingestion. Data showed significantly high mortality in flies with both injection and ingestion treatments after 24 h (F = 68.41, df = 3, P<0.05). However, no effect of sex on mortality related to infection was detected (F = 3.78, df = 1, P = 0.06) (Fig 3).

In order to monitor bacterial proliferation and ability to replicate in adult male and female flies, we measured the number of bacteria present in *C*. *capitata* at multiple time points after infection by injection. Flies wounded with a sterile needle didn’t show any presence of *Providencia* sp. There was a significant increase in the Log _10_ CFU of bacteria per fly (F = 167.36, df = 3, P<0.01). At 24 h post-infection, the bacterial load reached 11.29±2.3 Log_10_ CFU/FLY and 10.27±1.6 Log_10_ CFU/FLY in males and females respectively versus (5.40 ±0.07 and 5.17 ±0.07 Log_10_ CFU/FLY at 0h within males and females respectively). There was no sex-effect on bacterial load (F = 3.68, df = 1, P = 0.07). Males and females showed the same proliferation profile for *P*. *rettgeri* at all time points. Nonetheless, there is a significant correlation between mortality and bacterial load within infected females (R = 0.5, P = 0.02). This correlation is absent within infected males (R = -0.3, P = 0.4).

### Effect of mating status on mortality and bacterial proliferation

**Fig 4:**
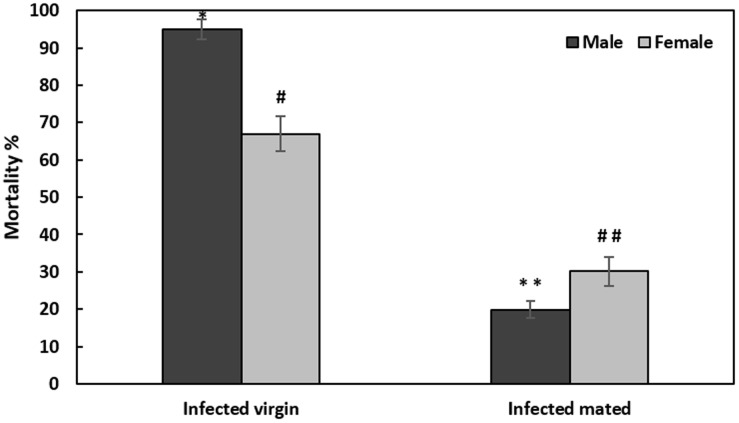
Effect of mating status and infection by injection with *Providencia rettgeri* on male and female *Ceratitis capitata* mortality. Virgin infected flies are flies from experiment “Infection by injection”. Each bar shows mean percentage± SE. Different symbols correspond to a significant difference (LSD test) for the males (*) and (#) for females.

We infected mated males and females 2 h after the end of copulation. A significant effect of mating on mortality was observed 24 h after infection (F = 18.91, df = 1, P = 0.001). The change in mortality after post-mating infection was significant for males and females. The mortality decreased to (19.91±2.12% versus 95±4.8 for infected virgin males. Whereas among females it decreased to 32.12 ±3.84% versus 88±4.2 for infected virgin females (Fig 4). Here the controls were infected virgin flies.

To estimate the possible effect of mating on bacterial infection, bacterial load was evaluated at two time points, after mating (0h and 24h). Bacterial load increased significantly over time after post-mating infection with *P*. *rettgeri* (F = 61.31, df = 3, P<0.01), to reach a maximum overall average at 24 h of 9.02± 0.56 Log_10_ CFU/FLY compared to 6.25±0.45 Log_10_ CFU/FLY at 0 h within males and 8.85±0.33 Log_10_ CFU/FLY at 24h compared to 6.5 ±0.33 Log_10_ CFU/FLY at 0h within females. Moreover, we noticed a significant sex-effect on bacterial load for *P*. *rettgeri* after mating (F = 5.30, df = 1, P = 0.03). Mortality in both mated males and females is significantly correlated to bacterial load at 24h (R = 0.86; P = 0.03, R = 0.61; P = 0.004, respectively).

### *Cecropin* gene relative density

**Fig 5:**
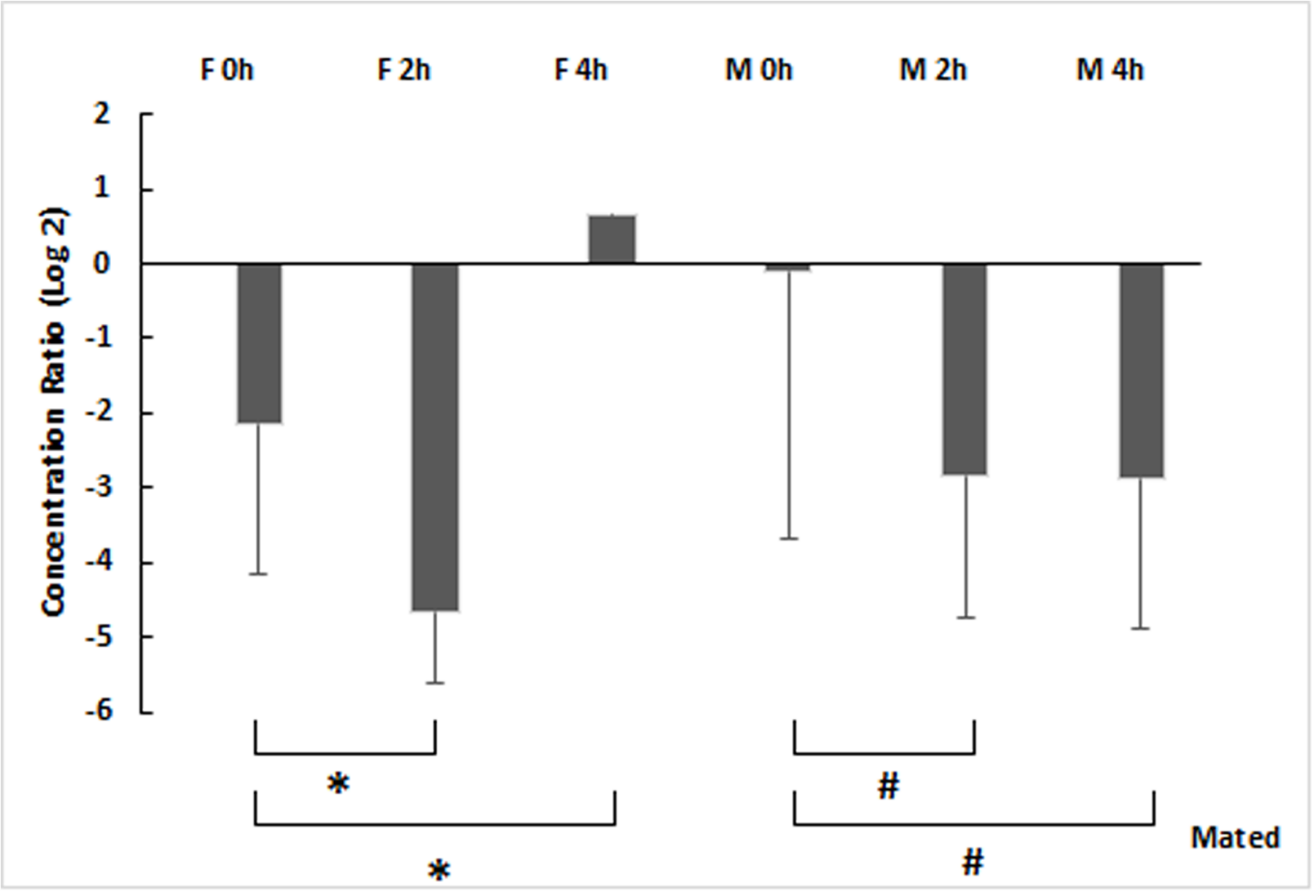
The relative density of cDNA amplicons of *Cecropin* gene expression (Log_2_ transformed fold) in infected post-mating *Ceratitis capitata* males and females compared to virgin ones. Each bar shows mean ± SE. Different symbols correspond to a significant difference, (*) for the females (F) and (#) for the males (M), (Multivariate analysis, MANOVA).

The fold induction of the *Cecropin* transcripts was calculated for the infected mated males and females compared to the virgin ones. Within mated females, the *Cecropin* is significantly underexpressed at 2h (5-fold) to be upregulated at 4h (Fig 5). Likewise, for males the transcripts abundance is significantly down-regulated at 2h (3-fold) and at 4h (3-fold) (F = 18, df = 2, P<0.01). Apart time, *Cecropin* regulation is substantially dependent of mating status (F = 8.76, df = 1, P<0.01). According to multivariate analysis, there is a significant interaction between mating status and time (F = 5.4, df = 1, P = 0.029).

## Discussion

This study aimed to isolate a strain of *Providencia* sp. from the digestive tract of the laboratory strain of *Ceratitis* “Vienna 8” destined for release in SIT programs worldwide. From the results of the numerical taxonomy of the 16S *rDNA*, this strain was identified as *Providencia rettgeri*, which belongs to the Enterobacteriaceae family. In line with numerous previous studies [51], our results confirmed the presence of bacteria of the genus *Providencia* in the insect’s gut and that *P*. *rettgeri* was a part of the natural gut flora of laboratory *Ceratitis* “Vienna 8” strain. However, it was unclear whether it was acting as a pathogen or was simply present in the natural environment. Although pathogenicity of *Providencia* sp. has been demonstrated in many insect orders and Nematodes, to our knowledge the virulence of *P*. *rettgeri* has only been shown particularly in *D*. *melanogaster* [32]. In the second part of our study, we determined the pathogenicity of *P*. *rettgeri* against *C*. *capitata*. As proposed by [32] pathogenicity is defined as the proportion of mortality caused by a bacterium and the bacterial ability to induce infection as measured by the expression of antimicrobial peptide (AMP) genes. Our results show that *P*. *rettgeri* could be virulent against *C*. *capitata*. Our medfly infection assays with *P*. *rettgeri* of both eggs and the adult stage showed significantly high levels of mortality and proportionally low levels of egg to pupae recovery.

The significant decrease in eggs that hatched after infection by an isolate of *P*. *rettgeri* recovered from the medfly Vienna 8 strain suggests that the bacteria crossed the eggshell and caused embryo death. Indeed, Lauzon et al., [51] demonstrated that after ingestion of *Enterobacter agglomerans* and *Klebstiella pneumonia* by medfly both bacterial strains colonized the apical end of developing and developed eggs and throughout all subsequent life stages. They observed bacteria within the micropyle and hypothesized that this could be an entry point into the egg before vertical transmission. Their observation may explain our results; *P*. *rettgeri* could have reached the embryos through the micropyle and was consequently transmitted to all the developing stages explaining the low pupal recovery. Under these circumstances, *Providencia* proliferation in the medfly leads to a quantitative reduction in the number of flies produced in the laboratory and egg to pupae recovery drops to unacceptable levels. One of the requirements of successful mass rearing for the SIT program is a continuous supply of a large number of excellent quality insects [13, 52, 53, 54].

The methodologies that have studied bacterial pathogenicity in insect species have used oral ingestion and injection of bacteria into the hemolymph. These two approaches induce more or less, different defense mechanisms. In our study, we used both injection in the hemolymph and ingestion of the bacteria in the lab strain insect, and we showed that virulence of *P*. *rettgeri* was similar in both cases. After introduction into the medfly through ingestion, *P*. *rettgeri* would cause a fatal septicemia after penetration through the insect’s gut wall and subsequent invasion of the hemocoel [55, 56].

*Providencia* has been reported to be a naturally occurring endosymbiotic bacterium in the digestive tract of medfly [15–22]. Nonetheless, the noted level of *Providencia* increases after irradiation to reach 22% among the total gut microbiota at 4 days [15]. On the other hand, the pathogenicity of *Providencia* sp. has been demonstrated against many insect orders and Nematodes and the virulence of *P*. *rettgeri* has been explicitly shown against other dipterans such as *D*. *melanogaster* [32, 57, 58]. Despite the natural prevalence of *P*. *rettgeri* in the digestive tract of the Mediterranean fruit fly, it can show harmful and pathogenic effects on its host. We noted that in the mortality assays there was a link between flies that showed a high bacterial load and the percentage of mortality at least within females. Whether or not there is a correlation between bacterial proliferation and host mortality may depend on the specific host-pathogen pair [32]. We can assume here using the two infection routes (ingestion and injection) that there was a tolerance with respect to *P*. *rettgeri* at low doses which was not the case at higher doses. The bacteria would acquire a pathogenicity revealed at higher microbial loads. This hypothesis is indeed supported by the egg infection experiment that showed that the egg hatching rate fell as the concentration of the bacterial suspension increased. The higher bacterial concentration was associated with higher mortality and consequently with lower egg-to-pupae recovery.

Therefore, we can suggest that the interaction of *Providencia* sp. with medfly should be classified as potential pathogenicity. Potential pathogens are defined by Onstad et al., [59] 1) microorganisms which have no method of invading or infecting a host but which can multiply and cause disease if they gain entrance, for example, through a wound; they grow readily in culture and do not cause specific diseases in specific hosts 2) a secondary invader such as *Serratia marcescens*. They are also defined as microorganisms that are incapable of invading the host either through the body wall or the digestive tract without the assistance of external factors that lower the insect’s resistance or enhance the ability of the microorganism to invade the host [60]. This latter definition seems to be plausible with our case because the rearing process and the irradiation render the medfly more sensitive [8, 9, 11, 14, 15]. Indeed, insect strains are subject to different constraints during laboratory rearing for many generations and the strains are kept for many years. Likewise, when they are mass produced for biocontrol purposes, as for establishing the Sterile Insect Technique (SIT) against medfly, it involves rearing huge numbers of flies for many generations under conditions that are very different from those in nature (colonization, adaptation processes, irradiation and prerelease handling). Additional disadvantages in fly quality can arise from long term inbreeding of colonies reared in highly artificial environments [61]. These conditions can result in inadvertent changes in the selection that acts on different physiological, biological and phenotypical aspects of males [12, 15, 62, 63, 64, 65, 66]. Nonetheless, the symbiotic bacteria associated with the medfly may play a significant role in these adaptations. Indeed, insect gut communities and particularly their role in fitness have recently been reviewed [67]. Several studies have been carried out on medfly by bacterial enrichment of the adult diet or simply by performing antibiotic treatments of the gut microbiota [19, 22, 22, 24, 68].

Populations of gut-associated microbiota in adult insects are tightly regulated and reflect a balance between the immune response and bacterial tolerance [69–70]. For both infection modes there was an immune response against *P*. *rettgeri* via anti-microbial peptides (AMPs) and the *Cecropin* gene expression was correlated to the bacteria proliferation and consequently to the mortality of the flies in both sexes.

After ingestion, there is a local secretion of AMPs which have an essential role in fighting oral infection by pathogenic bacteria. However, after direct injection into the body cavity there is a systemic immune defense against microorganisms [25]. In nature ingestion of microorganisms is probably the main route of infection, but bacteria can sometimes access the hemocoel directly either through an accidental breaching of the cuticle or by assisted transport via an entomophagous host. In our case, we found that both methods give the same results in terms of mortality and bacterial proliferation. The early work of Boman and colleagues [71] and all subsequent ones, showed that the method of infecting through a pinprick wound might mimic infections that wild flies can receive. But more recently, [58] working on *D*. *melanogaster* highlighted that the site of injury has an essential role for bacterial proliferation. They observed that thorax inoculation resulted in increased bacterial proliferation and caused high mortality within the first few days of infection. Indeed, our thorax infection with *P*. *rettgeri* induced high rates of mortality reaching almost 100% of males and females after only 24 h. In contrast abdomen inoculation resulted in minimal mortality and lower bacterial loads. However, they saw no difference in the induction of AMPs. The site of injury is thus an additional factor to be taken into account for further experiments.

While [40], [72] and [73] demonstrated that *D*. *melanogaster* females infected with a pathogenic bacterium suffered higher mortality if they had mated, in contrast after mating our results showed that mortality in males medfly was reduced five times, whereas in females medfly was reduced three times. Nevertheless, this reduction is still accompanied by an extensive bacterial proliferation at 24h. Indeed, the expression levels of *Cecropin* within mated males and females were significantly (compared to the virgins) but moderately down-regulated at 2h after infection (4.65-fold and 2.83-fold, respectively). This could explain the extensive proliferation of the bacteria until 24h. However, there is a significant increase of the transcripts at 4h (0.67-fold) within mated females compared to the virgin ones suggesting a modest resumption of the immune induction that could explain the mortality decrease. Therefore, it is useful to perform quantifications over a wider range of time and to increase the number of immune effectors to determine the responsibility of the high bacterial load and the mortality decrease in infected couples. *Cecropin* quantification alone is not sufficient to explain the findings.

Actually, Gomulski and colleagues [35] have shown that mating does not activate the immune gene expression for medfly. The post-mating immune response for *Cecropin 1* is moderately increased within males and females in abdomen and head parts. Whereas, other immune effectors such as *Defensin* and *Attacin* are, on the contrary, down-regulated. This slight increase in *Cecropin* is altered according to our results but not to the point of being able to affirm that there is a mating immunosuppressive effect, mostly if we consider mortality decrease. For *D*. *melanogaster*, mating is enriching the immune effectors expression in the absence of infection [72, 74, 75, 76] while, females are suffering from a post-mating lack of immune defense after infection [73]. The transfer of the seminal fluid components seems to be the primary cause of the alteration of the immune system activity within females forming a germline [73]. The eggless females are still immunocompetent after mating [73]. The survival test should have been carried out by an analysis of the seminal fluid components transferred to the mated and virgin females infected in order to elucidate the effect of the mating. The sperm abundance in association with the male accessory gland products is an additional factor that influences the immune response in infected females. However, within infected mated males, we might suppose that the decrease in the *Ceropin* levels until 4h is the cost of mating because of all these energetic investments of single male individuals to increase their mating frequency [77, 78, 79].

Finally, it is important to point out that a more thorough study of humoral defense, by the analysis of several effectors, and the cellular defense, is more than necessary, since their response might be mixed. The pathogen is also considered, because the mating effect seems to be pathogen-dependent due to microbial heterogeneity and not host dependence [38].

## Conclusion

In our work, we demonstrated that *P*. *rettgeri* was a part of the natural gut flora from laboratory *C*. *capitata*. However, under certain conditions it can present pathogenicity against *C*. *capitata*. The pathogenicity of *P*. *rettgeri* against *C*. *capitata* is dose-dependent. Thus, *P*. *rettgeri* can be considered as a potential pathogen of *C*. *capitata*. To date, there have been few studies on the complex associations between the medfly and its symbionts or on the molecular mechanisms of the insect’s immune response. The composition of intestinal microbiota is of prime importance for the fitness of sterile insects. Rearing stressors could impair this composition as is the case with the increase in the level of *Providencia* sp. or *Pseudomonas* sp. Enrichment with probiotics of the larval diet or at the adult stage as mentioned in recent studies could repair this imbalance and is recommended in insectaries. Virulence factors and toxins involved may provide a pathway for the development of biocontrol methods. All this requires improved knowledge of insect pathology.

## Supporting information

S1 Fig Summary of the methodology of medfly infection by *P*. *rettgeri*. (TIF)

S2 Fig Real time standard curve concentrations for *Cecropin* gene (A), *GAPDH2* gene (B) and *G6PD* gene (C). (DOCX)

S3 Fig Agarose gel electrophoresis detection of the PCR product from the 16S*rDNA*. (TIF)

S1 Table 16SrDNA, *Cecropin*, *GAPDH2* and *G6PD* full lengh and accession numbers. (DOCX)
