# Supplementary figures and images for: Evaluation of Providencia rettgeri pathogenicity against laboratory Mediterranean fruit fly strain (Ceratitis capitata)
Source: PLoS One. 2018 May 7;13(5):e0196343. doi: 10.1371/journal.pone.0196343 (PMC5937750; doi:10.1371/journal.pone.0196343)

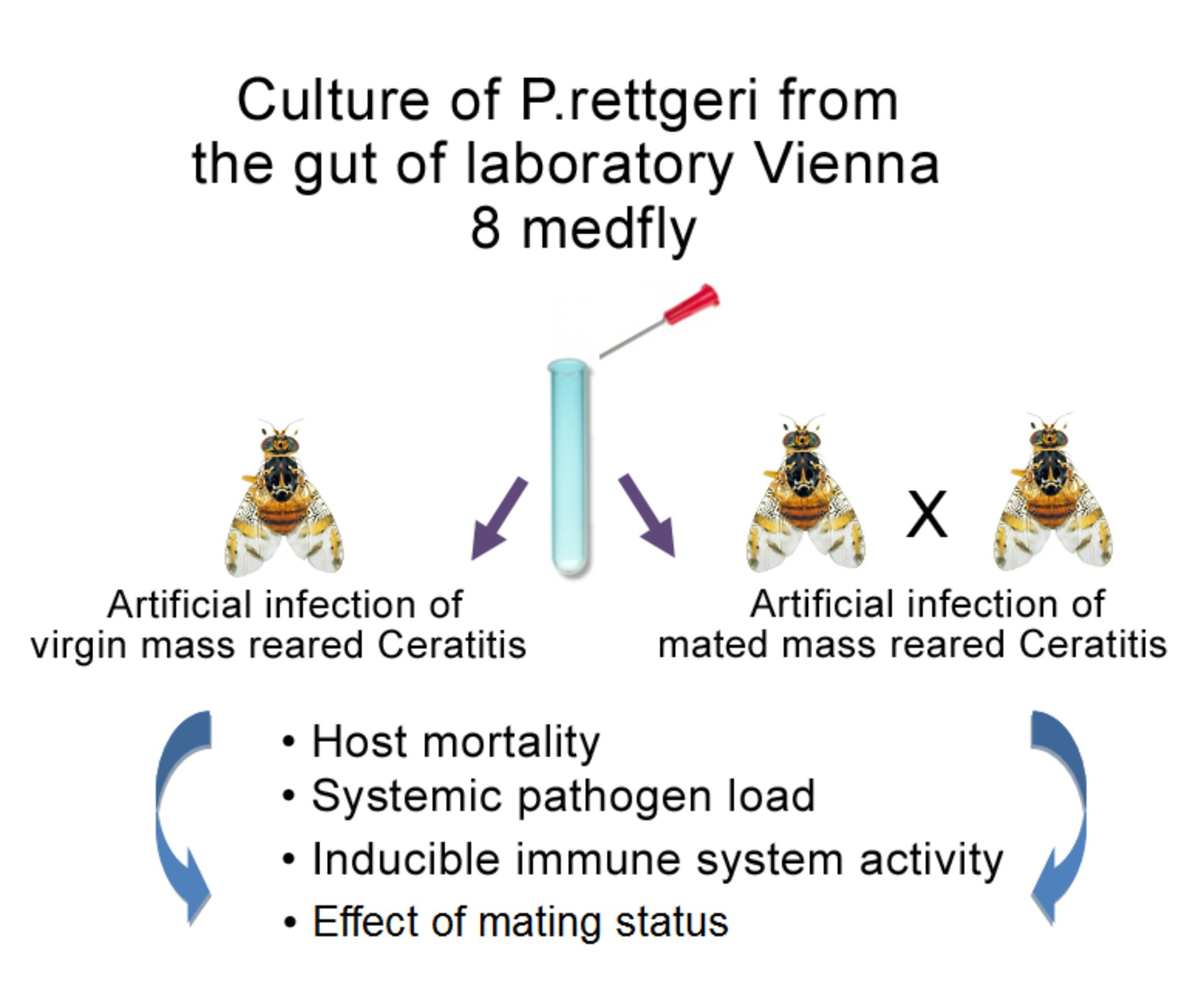

Supplement: S1 Fig — (TIF) [file pone.0196343.s001.tif]

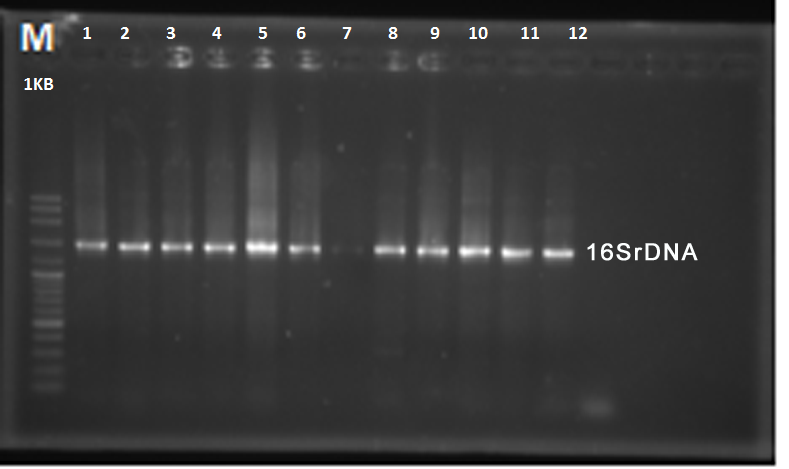

Supplement: S3 Fig — (TIF) [file pone.0196343.s003.tif]
