# Supplementary material for: Evaluation of Providencia rettgeri pathogenicity against laboratory Mediterranean fruit fly strain (Ceratitis capitata)
Source: PLoS One. 2018 May 7;13(5):e0196343. doi: 10.1371/journal.pone.0196343 (PMC5937750; doi:10.1371/journal.pone.0196343)
Supplement: S2 Fig — (DOCX) [file pone.0196343.s002.docx]

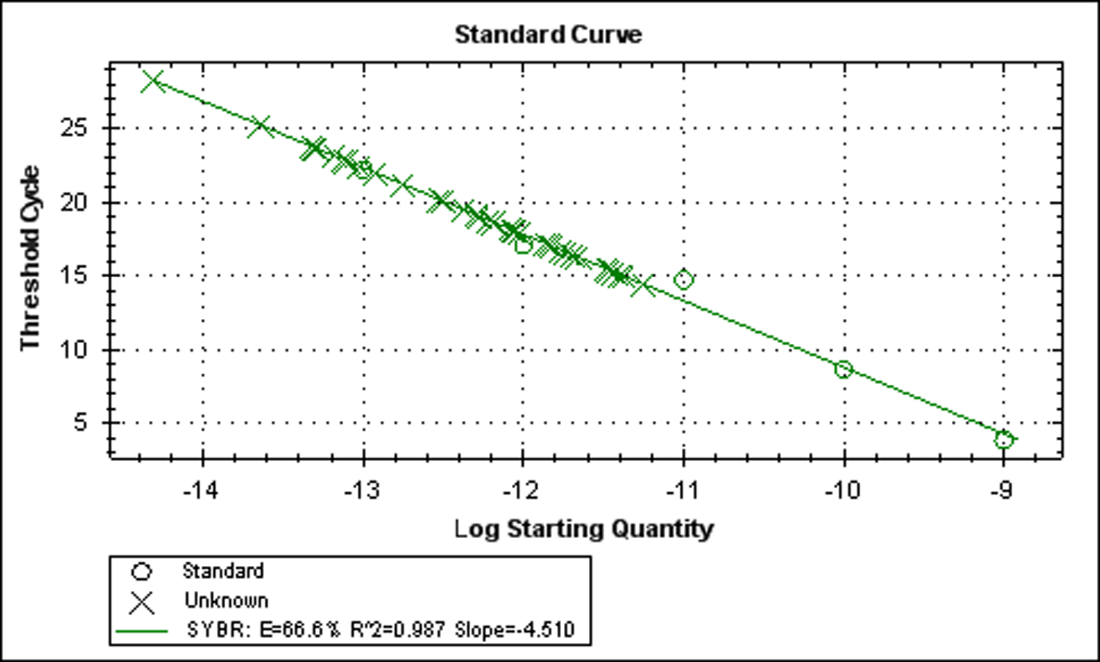


**S2 (A) Fig. Standard curve of Cecropin gene**

**
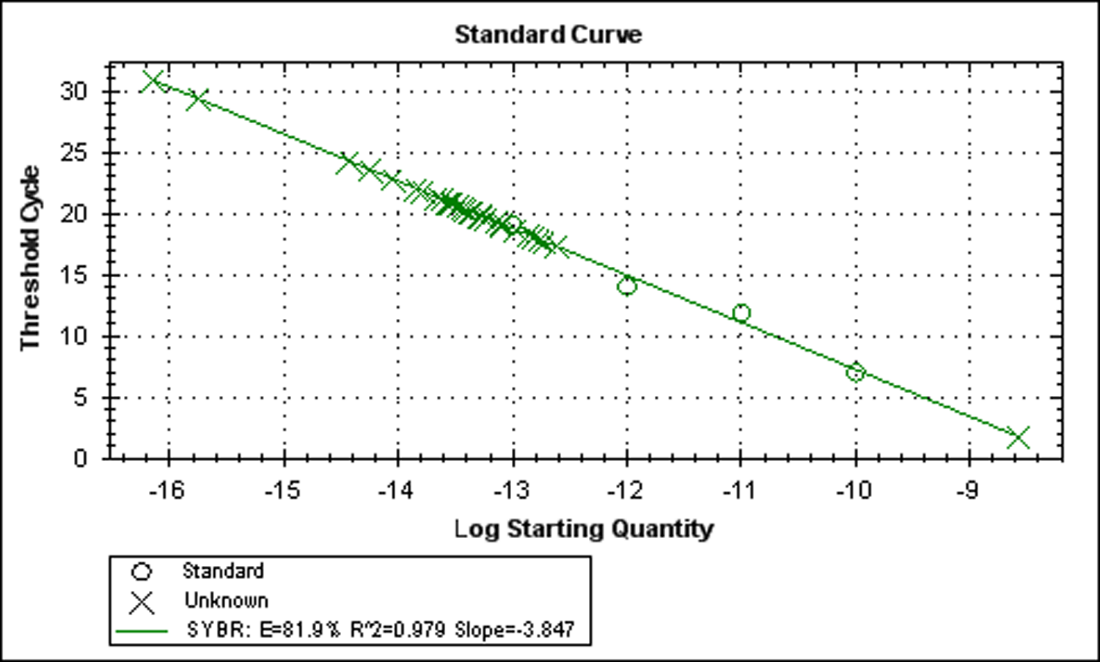
**

**S2 (B) Fig. Standard curve of GAPDH2 gene**

**
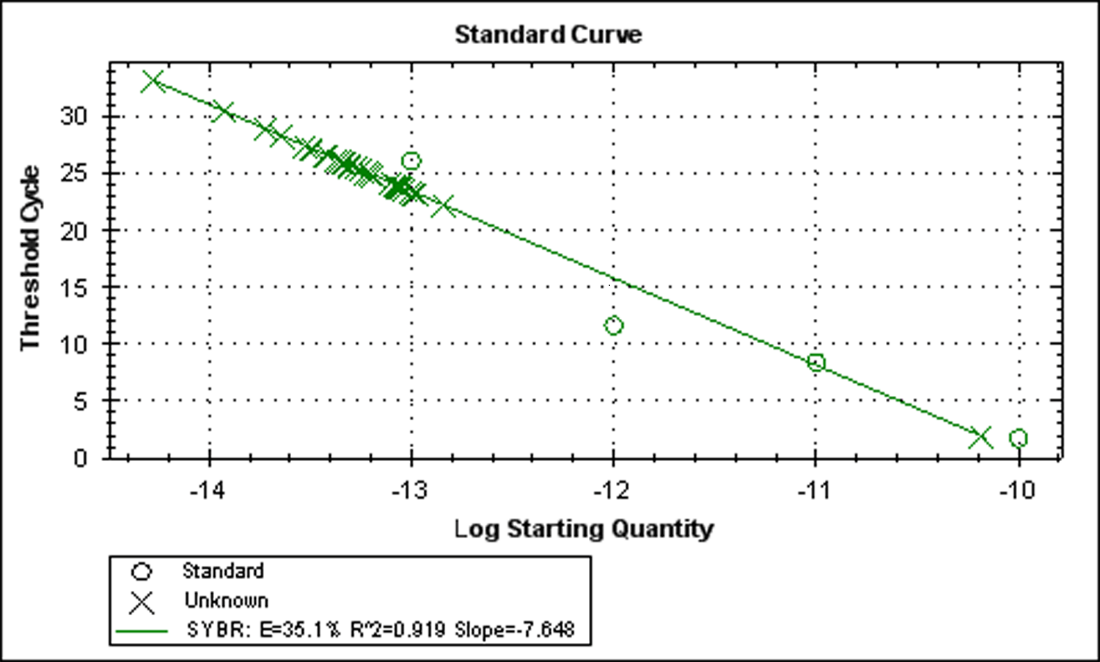
**

**S2 (C) Fig. Standard curve of G6PDH gene**
