# Supplementary material for: Evaluation of Providencia rettgeri pathogenicity against laboratory Mediterranean fruit fly strain (Ceratitis capitata)
Source: PLoS One. 2018 May 7;13(5):e0196343. doi: 10.1371/journal.pone.0196343 (PMC5937750; doi:10.1371/journal.pone.0196343)
Supplement: S1 Table — (DOCX) [file pone.0196343.s004.docx]

| **Gene** | **Primer refrence [ ]** | **Accession number** |
| --- | --- | --- |
| 16S *rDNA* | 5’ agagtttgatcctggctcag 3’ (Forward primer)  5’ ctacggctaccttgttacga 3’ (reverse primer) [1] | MG752971 |
| *Cecropin 1* | 5’ gcgggttggctgaagaag 3’ (Forward primer)  5’ cggtggctgcgacattag 3’ (Reverse primer) [2] | X70030 |
| *GAPDH2* | 5 ‘ggtcgcatcggtcgtctgg 3’ (Forward primer) 5’ gctgaaacggtgcccttgaaac 3’ (Reverse primer) [2] | FS831 |
| *G6PDH* | 5’ cggacgagcaggcaaaatatg 3’ (Forward primer)  5’ agacggacggcggtaagg 3’ (Reverse primer) [2] | S67872 |

[1] Daffonchio D, Borin S, Frova G, Manachini P, Sorlini C. PCR fingerprinting of whole genomes: the spacers between the 16S and *23S rRNA* genes and of intergenic *tRNA* gene regions reveal a different intraspecific genomic variability of Bacillus cereus and Bacillus licheniformis. Intern J Syst Bacteriol. 1998; 48, 107- 16.

[2] Vandesompele J, De Preter K, Pattyn F, Poppe B, Van Roy N. Accurate normalization of real-time quantitative RT-PCR data by geometric averaging of multiple internal control genes. Genome Biology. 2002; 3(7): RESEARCH0034.1-0034.11.
